# Research on design strategy of one-piece ski suit driven by demand

**DOI:** 10.1038/s41598-026-35593-4

**Published:** 2026-01-17

**Authors:** Xiangdong Luo, Zhetao Zhang, Wei Qiang, Meng Wu

**Affiliations:** Shaanxi University of Science and Technology, Xi ’an, 710021 Shaanxi Province China

**Keywords:** One-piece ski suit, User requirements, User journey map, Kano-AHP, Human behaviour, Psychology and behaviour, Socioeconomic scenarios

## Abstract

In order to effectively obtain the design requirements and weights of users when wearing one-piece ski suits, and at the same time determine and quantify them into design strategies based on the requirements, so as to effectively improve user satisfaction and enhance user wear experience. This research first constructs a user journey map through user interviews, analyzes the behavioral paths of users when wearing ski suits and sorts out user needs; secondly, creates a Kano five-order two-factor questionnaire to investigate the user’s design needs evaluation of one-piece ski suits, and uses the Kano model to analyze the characteristics of user needs; thirdly, use the AHP hierarchical analysis method to construct a one-piece ski suit design needs analysis model, and proposes a one-piece ski suit design strategy based on calculating the user demand weights at each level, and finally tests the rationality of the method through design cases. The research results show that by establishing a method of obtaining user needs with Kano-AHP, the importance of one-piece ski suit design elements is sorted and quantified, and a design strategy to improve user satisfaction is proposed more scientific and reasonable, providing new methods and ideas for related design research.

## Introduction

 According to the website of the Central People’s Government of China, as of October 2021, the number of people participating in ice and snow sports nationwide reached 346 million, achieving the goal of “driving 300 million people to participate in ice and snow sports” ahead of schedule^1^. With the successful hosting of the Winter Olympic Games in 2022, ski resorts have been built all over the country, greatly promoting the popularization and development of skiing. Skiing in China is gradually changing from a niche sport to a popular winter sport. The Opinions of the General Office of the CPC Central Committee on Taking the 2022 Beijing Winter Olympics as an Opportunity to Vigorously Develop Ice and Snow Sports made arrangements to accelerate the development of ice and snow industry and supported enterprises to develop ice and snow sports products with high scientific and technological content and independent intellectual property rights^2^. Several Opinions of the General Office of the State Council on Stimulating the Economic Vitality of Ice and Snow with High-quality Development of Ice and Snow Sports pointed out that measures to stimulate the economic vitality of ice and snow through the development of ice and snow sports especially emphasized the promotion of high-quality development of ice and snow equipment industry, striving to improve equipment research and development capability and independent manufacturing level, and promoting the modernization of the entire ice and snow equipment industry chain^3^. Ski clothing is a necessary equipment in skiing, which can provide sports protection while ensuring comfortable wearing. It is designed to meet the principles of light weight, warmth, wind and snow protection, comfortable fit, and does not hinder movement. It also has anti-wear, anti-stretch and anti-tear properties to adapt to complex sports environments^4,5^.

At present, the research on ski clothing mainly focuses on two aspects: (1) taking ski clothing products as the core. It mainly focuses on the performance, comfort and functionality of clothing, including clothing structure design and clothing material research. For example, Feng Mingming et al.^6^ conducted a study on the influence of the air layer under the clothing on the thermal and wet comfort of ski clothing using new biomass graphene inner warm fleece as thermal insulation material. Yuan Weiwei et al.^4^ screened two excellent fabrics through performance test and factor analysis, which were used to make ski suits and evaluate their performance in key movements. Liu Dan et al.^7^ used SST k-ω turbulence model and CFD method to predict the aerodynamic characteristics of five different ski jumping suits in flight phase under the condition of surface roughness. Wang J et al.^8^ designed 6 kinds of jacquard knitted fabrics and knitted them with polyester and DRYARN materials. Through comprehensive performance evaluation and wearing experiment, the excellent functionality of seamless ski underwear knitted based on human sportswear was verified. Toolis T et al.^9^ evaluated the impact of wearing upper-body and lower-body compression clothing on the cross-country skiing performance of elite biathlon athletes. (2) Guided by user needs. By means of requirement analysis model and statistical method, user’s design requirements are obtained and relevant design strategies are put forward. For example, Liu Jing et al.^10^ studied the dress evaluation of snowboarders and their requirements in terms of structure, style and function. Chen Yanrong^11^ put forward the design method of snowboarding clothing by analyzing the user requirements centered on drop resistance and skiing posture. Kim K^12^ identified functional design element requirements for children’s ski wear through functional evaluation. Kwon et al.^13^ analyzed the impact of men’s ski wear sizing system on purchasing behavior. Wu J et al.^14^ applied the human-oriented design method to explore the development strategy of intelligent sportswear for sitting skiers.

Through literature review, it can be known that at present, the overall number of studies on ski clothing is relatively small, and most of the existing studies are focused on the ski clothing products themselves, with only a few studies discussing user needs. Few scholars have studied the importance of design elements of one-piece ski suits based on the demand model and proposed design strategies to improve the wearing experience of users. Therefore, taking the design of one-piece ski suits as the research object and through the Kano-AHP method, studying how to drive the design strategy of one-piece ski suits from the perspective of meeting user needs has certain practical value.

## Construction of the demand discovery model for one-piece ski suits

With the advancement of science and technology and the vigorous development of the ice and snow industry, new product development has become an effective means for ski clothing enterprises to enhance their market competitiveness. Against the backdrop of increasingly diverse and complex ski clothing design, conducting user demand analysis before design is not only a prerequisite for ensuring the effectiveness of design and development, but also a key reference basis throughout the entire design and development process^15^.

In the design of one-piece ski suits oriented by user demands, the Kano model is a tool specifically designed for classifying user demands and prioritizing them. It is a qualitative analysis method for analyzing the characteristics of design demands based on the impact of design demands on user satisfaction^16^. When the original demands of users are numerous and ambiguous, using the Kano model to distinguish the demand categories can optimize the wearing experience of users in a targeted manner and allocate design resources more effectively. The specific operation is: Firstly, the Kano questionnaire was used to survey users’ evaluations of the original demands. Then, through analysis, user needs are divided into five types: Must-be Quality (M): The absence will significantly reduce user satisfaction, but its realization will not improve it; One-dimensional Quality (O): Its realization will improve user satisfaction, while its absence will reduce it; Attractive Quality (A): Its realization will bring surprise and a sharp rise in satisfaction, and its absence has no impact; Indifferent Quality (I): Whether it exists or not has no impact on satisfaction; Reverse Quality (R): Its realization will instead lead to a decrease in user satisfaction. Finally, the design requirement priority of the one-piece ski suit is obtained by calculating the Better-Worse coefficient of each requirement. The Kano model provides directional support for the construction of design strategies^17^, but the Kano model has limitations in presenting and comparing the importance of requirement elements at different levels and the same level^18^.

The AHP method is a quantitative analysis approach that determines the importance of each factor by constructing a hierarchical model and expert decision-making based on in-depth analysis of complex decision-making problems^19,20^. The specific operation is as follows: First, a hierarchical structure model is constructed to subdivide user requirements into three levels: the objective level, the criterion level, and the sub-criterion level. Then, a judgment matrix is built for expert scoring. Finally, the weights of each indicator are calculated and consistency checks are conducted. The AHP method provides decision-makers with the data support needed to evaluate and compare different solutions, thereby effectively enhancing the scientificity and rationality of the design process.

**Fig. 1:**
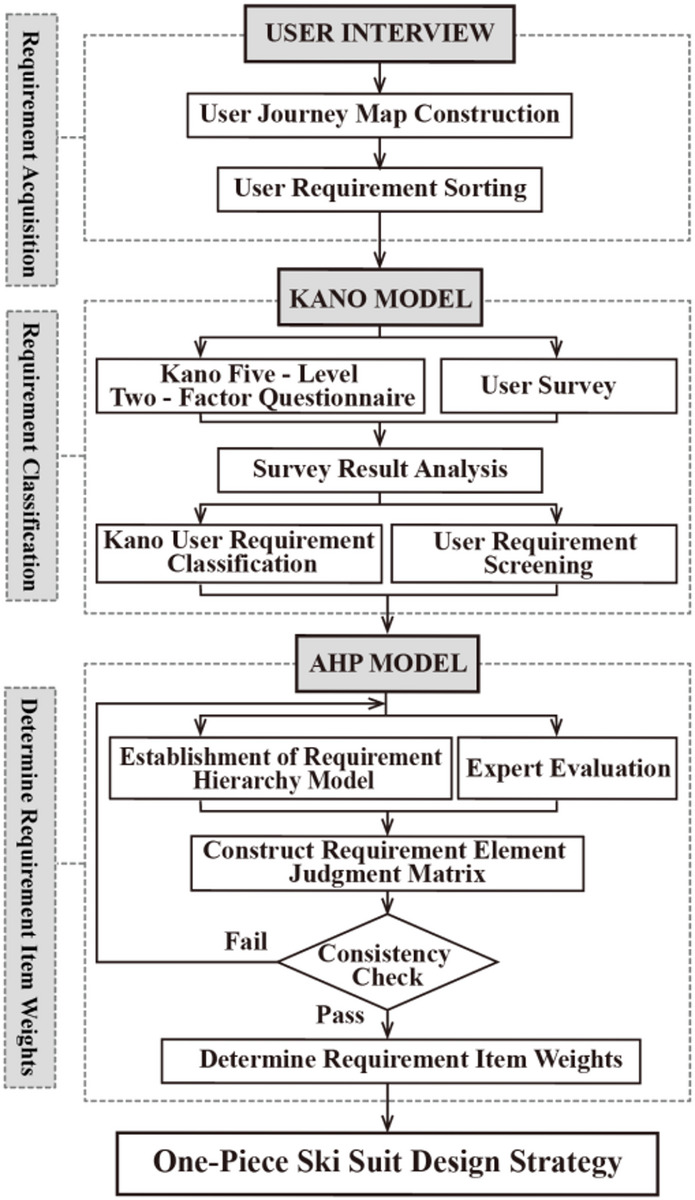
Design model of one-piece ski suits based on user needs.

Both the Kano model and the AHP method have solid theoretical foundations in the fields of demand analysis and decision analysis. The method combining qualitative and quantitative analysis aims to handle complex decision-making situations with multiple objectives and criteria, in order to enhance the rationality and accuracy of the decision-making process^21^. The Kano model effectively reveals the multi-level complexity of user requirements by distinguishing design requirements. Meanwhile, the AHP rule provides decision-makers with a comprehensive system analysis framework by adopting a hierarchical structure model and pairwise comparison. These two methods jointly provide strong theoretical support and practical tools for understanding user needs and optimizing the decision-making process. The innovation of this study lies in the combination and application of these two methods to the design of one-piece ski suits, which has not been widely explored in the existing literature. The research first identifies the core needs of skiing participants through the Kano model, and then prioritizes these needs using the AHP method. Combining these two methods, not only can the requirements be identified, but also their impact on user satisfaction can be quantified, thereby providing more precise decision support for the evolutionary design strategy of this clothing. The design model of the one-piece ski suit based on user requirements is shown in Fig. 1.

## Design requirement analysis based on the Kano model

### Acquisition of user requirements

The user journey map is an analytical tool often used by designers to reveal user pain points and refine user needs^22^. It divides the interaction behaviors of one-piece ski suit users with the ski suit during skiing activities into different stages. Firstly, investigate the behavioral paths of users wearing one-piece ski suits. Secondly, based on the satisfaction degree of the wearers with all contact points and the pain points in the wearing experience during this process, transform them into the original design requirements of users for the design of one-piece ski suits^23^.

**Fig. 2:**
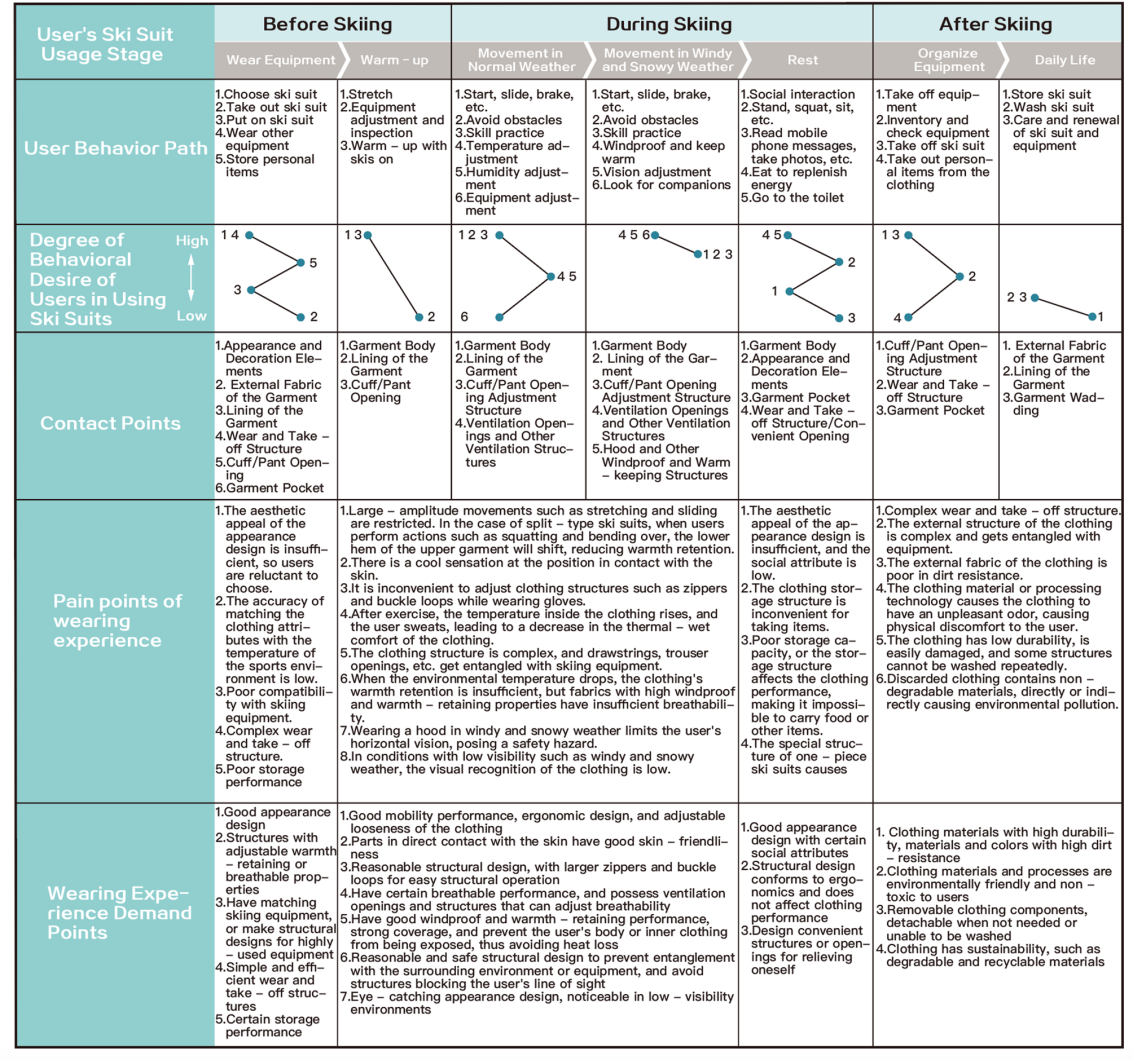
User journey map of one-piece ski suits.

Due to the particularity of the one-piece ski suit design, its main purpose is to be the sports clothing worn by users during skiing. Therefore, taking the entire process of skiing as the research path of user design requirements has certain practical significance. This survey was conducted in an age group with a relatively high number of skiers and a balanced gender ratio. The surveyed subjects are aged 18–40 and all have relatively rich skiing experience. The research was carried out by visiting 2 professional ski equipment enterprises and 1 professional ski resort. The total number of respondents is 30, including 16 males and 14 females, among whom there are 4 ski suit designers, 2 professional skiers, 7 amateur skiers, and 17 senior skiing enthusiasts. When engaging in skiing, the process of the wearer routinely using ski suits mainly consists of three stages: putting on the equipment before skiing and warming up; Normal weather sports in skiing, sports in snowy and windy weather, and rest; After skiing, packing up equipment and daily life. The user behavior paths at each stage were obtained through questionnaires and interviews. The behaviors of users at each stage and the contact points with ski clothing were summarized and listed. The pain points and wearing experience demand points were extracted and recorded in the form of a user journey map, as shown in Fig. 2.

User feedback on ski suits during skiing was collected through the user journey map, and user design requirements were sorted out by combining the involved fashion design elements. According to the terms and definitions in the National Standard of the People’s Republic of China *Professional sportswear—Ski and snowboard wear* (GB/T 41176 − 2021)^24^, and combined with the expert suggestions from 2 professional skiwear designers, the user requirements for one-piece ski suits were further summarized into 24 items in total, divided into five categories: basic performance, structural design, appearance design, safety performance, and sustainability. The classification results are shown in Table 1.

**Table 1:** Classification of design requirements for one-piece ski suits.

Number	Needs classification	Needs Items	Number	Needs Classification	Needs Items
R1	Basic performance	Effective thermal insulation design	R15	Appearance Design	Aesthetic visual design
R2		Effective wind resistance design	R16		Prominent brand identity design
R3		Comprehensive coverage design	R17		Stain-resistant color scheme design
R4		High flexibility design	R18	Safety Performance	Safety warning design in appearance
R5		Effective breathability design	R19		Basic protective design (abrasion resistance, cut resistance, etc.)
R6		Multiple storage pockets design	R20		Low snagging potential in garment structure
R7		Skin-friendly lining design	R21		Non-obstructive vision design
R8	Structural design	Adjustable ventilation openings design	R22	Sustainability	High durability of garment materials
R9		Adjustable tightness design	R23		Garment materials harmless to the human body
R10		Detachable component/liner design	R24		Use of regenerated sustainable materials
R11		Enlarged zipper and fastener design			
R12		Ergonomic functional layout for convenient operation			
R13		Easy on-and-off design			
R14		Convenient access openings design			

### Kano questionnaire design

To establish the relationship between the design requirements of one-piece ski suits and user satisfaction and classify the requirement attributes, the Kano model questionnaire was designed based on the initial user requirements obtained from the previous research. The questionnaire is mainly divided into two parts. The first part is the basic information, collecting the gender, age and skiing experience of the respondents to ensure that they have sufficient wearing experience of ski suits to guarantee the validity of the questionnaire. The second part adopts the form of a five-point scale and asks questions about the same requirement from two dimensions: positive and negative, that is: what are users’ attitudes if the requirement item is available and unavailable respectively. It aims to identify the asymmetric impact of the existence and absence of functions on user satisfaction, so as to achieve the accurate classification of requirement attributes. The attitude options in the scale are divided into 5 dimensions: Like: It brings surprise when provided, and there is no dissatisfaction when not provided; Expected: It is taken for granted when provided, and there is strong dissatisfaction when not provided; Indifferent: Whether it is provided or not has no impact on satisfaction; Tolerable: It is tolerable when not provided, but there is dissatisfaction when the provision is poor; Dislike: It instead causes dissatisfaction when provided. The questionnaire design is shown in Table 2.

To ensure the validity of the questionnaire, corresponding explanations are provided in the questionnaire for positive and negative bipolar questions that may cause obstacles to respondents’ answering, options of the Likert scale, and requirement items with strong professionalism.

**Table 2:** Kano two-factor five-level likert questionnaire.

User Requirements for One-Piece Ski Suits	Question setting for two-way questioning (choose one answer for each question)
R1: Effective thermal insulation design	How do you feel if the R1 element appears in the design of the one - piece ski suit? □ Like □ Expected □ Indifferent □ Tolerable □ Dislike
	How do you feel if the R1 element doesn’t appear in the design of the one - piece ski suit? □ Like □ Expected □ Indifferent □ Tolerable □ Dislike
R2: Effective wind resistance design	How do you feel if the R2 element appears in the design of the one - piece ski suit? □ Like □ Expected □ Indifferent □ Tolerable □ Dislike
	How do you feel if the R2 element doesn’t appear in the design of the one - piece ski suit? □ Like □ Expected □ Indifferent □ Tolerable □ Dislike
R3:Comprehensive coverage design	How do you feel if the R3 element appears in the design of the one - piece ski suit? □ Like □ Expected □ Indifferent □ Tolerable □ Dislike
	How do you feel if the R3 element doesn’t appear in the design of the one - piece ski suit? □ Like □ Expected □ Indifferent □ Tolerable □ Dislike
R4:High flexibility design	How do you feel if the R4 element appears in the design of the one - piece ski suit? □ Like □ Expected □ Indifferent □ Tolerable □ Dislike
	How do you feel if the R4 element doesn’t appear in the design of the one - piece ski suit? □ Like □ Expected □ Indifferent □ Tolerable □ Dislike
……	……

### Questionnaire analysis

This survey conducted offline surveys on users at ski resorts and ski equipment stores. According to the design idea of the questionnaire, the main target group of this questionnaire survey was people aged 18 to 40 who had the experience of wearing ski suits, and a total of 203 electronic questionnaires were distributed. After eliminating the invalid questionnaires with abnormal answer information, 190 valid questionnaires were obtained, and the effective rate of the questionnaires was 94%. Through the user attitude survey of 24 original requirements for one-piece ski suits, the demand evaluation forms were summarized. Classified according to the quality characteristics of Kano, the attribute categories of each design requirement were clarified.

Taking the design requirement item of “R1 effective thermal insulation design” as an example, based on the results of users’ responses to positive and negative questions in the Kano questionnaire, the category with the highest frequency of occurrence is determined as the final attribute of this requirement according to the evaluation criteria in the form of Table 3^25^. Among them, the proportion of Must-be Quality (M) is the sum of response proportions where users “dislike it when it is unavailable” and “only express ‘Expected’, ‘Indifferent’ or ‘Tolerable’ when it is available”: 20.20% + 40.39% + 23.15% = 83.74%; the proportion of Attractive Quality (A) is the sum of response proportions where users “have no negative attitude when it is unavailable but like it when it is available”: 1.97% + 2.46% + 1.48% = 5.91%; the proportion of One-dimensional Quality (O) is the cell where users “dislike it when it is unavailable and like it when it is available”: 1.48%; the proportion of Indifferent Quality (I) is the sum of proportions where attitudes are all neutral: 0.99% + 2.96% + 1.97% + 1.48% + 1.48% = 8.87%; there are no Reverse Quality or Conflict Response. Data show that among the surveyed users, the proportion of those who consider it a Must-be Quality (M) requirement is the highest, at 83.74%; therefore, the one-piece ski suit design requirement item of “R1 effective thermal insulation design” is classified as a Must-be Quality (M) requirement.

Questionnaires for the remaining 23 design requirement items were counted in accordance with the above-mentioned form, and the classification results obtained from the division of design requirement attributes for one-piece ski suits are shown in Table 5.

**Table 3:** Comparison table of Kano model evaluation results classification of R1 requirements.

Requirement R1: Effective thermal insulation design						
Available/Not available		Reverse question: How do you feel if the R1 element doesn’t appear in the design of the one - piece ski suit? (Percentage of people)				
		like	Expected	Indifferent	Tolerable	dislike
Positive question: How do you feel if the R1 element appears in the design of the one - piece ski suit? (Percentage of people)	like	Q	A (1.97%)	A (2.46%)	A (1.48%)	O (1.48%)
	Expected	R	I	I (0.99%)	I (2.96%)	M (20.20%)
	Indifferent	R	I (1.97%)	I (1.48%)	I	M (40.39%)
	Tolerable	R	I	I	I (1.48%)	M (23.15%)
	dislike	R	R	R	R	Q
Total	M (83.74%)	O (1.48%)	A (5.91%)	I (8.87%)	R (0%)	Q (0%)

Note: Q: Conflict Response (Exclusion); M: Must-Be Quality; O: One-Dimensional Quality; A: Attractive Quality; I: Indifferent Quality; R: Reverse Quality.

Reliability and validity tests of the Kano questionnaire were conducted using SPSS statistical software, and the reliability and validity analysis of the Kano questionnaire is shown in Table 4. The Cronbach’s α value of the overall Kano questionnaire is 0.810 > 0.8; among them, the value for positive questions is 0.757, and the value for reverse questions is 0.728, both of which are between 0.7 and 0.8. This indicates that the questionnaire in this study has good reliability. For the validity test, the KMO measure value is 0.765, which is between 0.7 and 0.8, indicating that it is relatively suitable for factor analysis. In addition, the significance probability of the Bartlett’s Test of Sphericity statistic is 0.000 < 0.01, indicating that the data is suitable for factor analysis and has correlation. The cumulative variance contribution rate is 72.532% > 60%, indicating that the questionnaire has good construct validity and is suitable for Kano model analysis.

**Table 4:** Reliability and validity analysis of the Kano Questionnaire.

Item		Value
Cronbach’s α Value	All Questions	0.810
	Positive Questions	0.757
	Reverse Questions	0.728
KMO Measure		0.765
Bartlett’s Test of Sphericity	Sig.	0.000
Cumulative Variance Contribution Rate		72.532%

In order to obtain users’ design requirements more accurately and enable the final designed clothing to have higher user satisfaction, the Better-Worse coefficient was calculated to quantify the impact of the original design requirements of one-piece ski suits on user satisfaction^26^. It includes the satisfaction coefficient (Better coefficient) and the dissatisfaction coefficient (Worse coefficient). The closer the value of the Better coefficient is to 1, the more it indicates that meeting the demand can significantly increase the user’s satisfaction. The closer the value of the Worse coefficient is to −1, the more it indicates that the failure to meet this requirement will significantly reduce the user’s satisfaction^27^. The calculation formula for the better-worse indicator is as follows.

The Better coefficient is shown in Eq. (1) :

1 $$\:B=\frac{A+O}{A+O+M+I}$$

The Worse coefficient is shown in Eq. (2) :

2 $$\:W=\frac{O+M}{A+O+M+I}\times\:(-1)$$

Using the SPSS statistical software, based on the proportion of the respondents’ choices of categories A, M, O, I, R in each demand, the values of the Better-Worse coefficients were calculated respectively according to formulas (1) and (2). Take the demand item of “R1 effective thermal insulation design” as an example. Through calculation, the Better coefficient is 7.39% and the Worse coefficient is −85.22%. The selection results of the remaining 23 design requirements are calculated according to the above method to obtain the Better-Worse coefficient of each requirement item.

The attribute classification results of the 24 original design requirements of users for ski suit design and the calculation results of the Better-Worse coefficient are shown in Table 5.

**Table 5:** Analysis result table of the Kano model.

Needs Items	A	O	M	I	*R*	Q	Attribute classification result	Better	Worse
R1	5.91%	1.48%	83.74%	8.87%	0.00%	0.00%	M	7.39%	−85.22%
R2	4.93%	3.45%	81.77%	9.85%	0.00%	0.00%	M	8.37%	−85.22%
R3	3.94%	47.78%	9.85%	23.15%	15.27%	0.00%	O	61.05%	−68.02%
R4	14.29%	46.80%	12.32%	21.67%	4.93%	0.00%	O	64.25%	−62.18%
R5	8.87%	12.32%	52.71%	26.11%	0.00%	0.00%	M	21.18%	−65.02%
R6	17.24%	12.81%	13.79%	39.41%	16.75%	0.00%	I	36.09%	−31.95%
R7	35.96%	7.88%	12.32%	28.57%	15.27%	0.00%	A	51.74%	−23.84%
R8	55.67%	3.45%	7.88%	19.70%	13.30%	0.00%	A	68.18%	−13.07%
R9	6.40%	5.42%	9.85%	41.87%	36.45%	0.00%	I	18.60%	−24.03%
R10	3.94%	3.94%	7.88%	26.60%	57.64%	0.00%	R	18.60%	−27.91%
R11	35.47%	10.34%	5.42%	32.51%	16.26%	0.00%	A	54.71%	−18.82%
R12	19.70%	39.41%	28.08%	12.81%	0.00%	0.00%	O	59.11%	−67.49%
R13	53.69%	17.24%	20.69%	8.37%	0.00%	0.00%	A	70.94%	−37.93%
R14	12.81%	51.23%	10.84%	17.24%	7.88%	0.00%	O	69.52%	−67.38%
R15	49.75%	1.97%	23.65%	24.63%	0.00%	0.00%	A	51.72%	−25.62%
R16	4.43%	4.93%	11.82%	44.83%	33.99%	0.00%	I	14.18%	−25.37%
R17	1.48%	2.46%	3.45%	41.38%	51.23%	0.00%	R	8.08%	−12.12%
R18	13.30%	34.98%	16.26%	23.65%	11.82%	0.00%	O	54.75%	−58.10%
R19	7.39%	1.97%	74.38%	14.78%	1.48%	0.00%	M	9.50%	−77.50%
R20	2.96%	5.91%	18.23%	55.17%	17.73%	0.00%	I	10.78%	−29.34%
R21	47.78%	2.96%	5.91%	33.50%	9.85%	0.00%	A	56.28%	−9.84%
R22	3.94%	1.48%	34.48%	48.77%	11.33%	0.00%	I	6.11%	−40.56%
R23	25.12%	33.50%	17.24%	20.69%	3.45%	0.00%	O	60.71%	−52.55%
R24	3.94%	2.46%	4.93%	19.21%	69.46%	0.00%	R	20.97%	−24.19%

Note: Q: Conflict Response (Exclusion); M: Must-Be Quality; O: One-Dimensional Quality; A: Attractive Quality; I: Indifferent Quality; R: Reverse Quality.

It can be known from Table 5 that the Kano types of 24 original design requirement indicators for ski suit design have been obtained through Kano analysis and calculation. Among them, there are 4 must-be quality requirement index (M), namely: R1, R2, R5, and R19; there are 6 one-dimensional quality requirement index (O), namely: R3, R4, R12, R14, R18, and R23; there are 6 attractive quality requirement index (A), namely R7, R8, R11, R13, R15 and R21. In addition, there are 5 indifferent quality requirement index (I), namely: R6, R9, R16, R20, and R22; there are 3 reverse quality requirement index (R), namely: R10, R17, and R24, none of which will be considered.

**Fig. 3:**
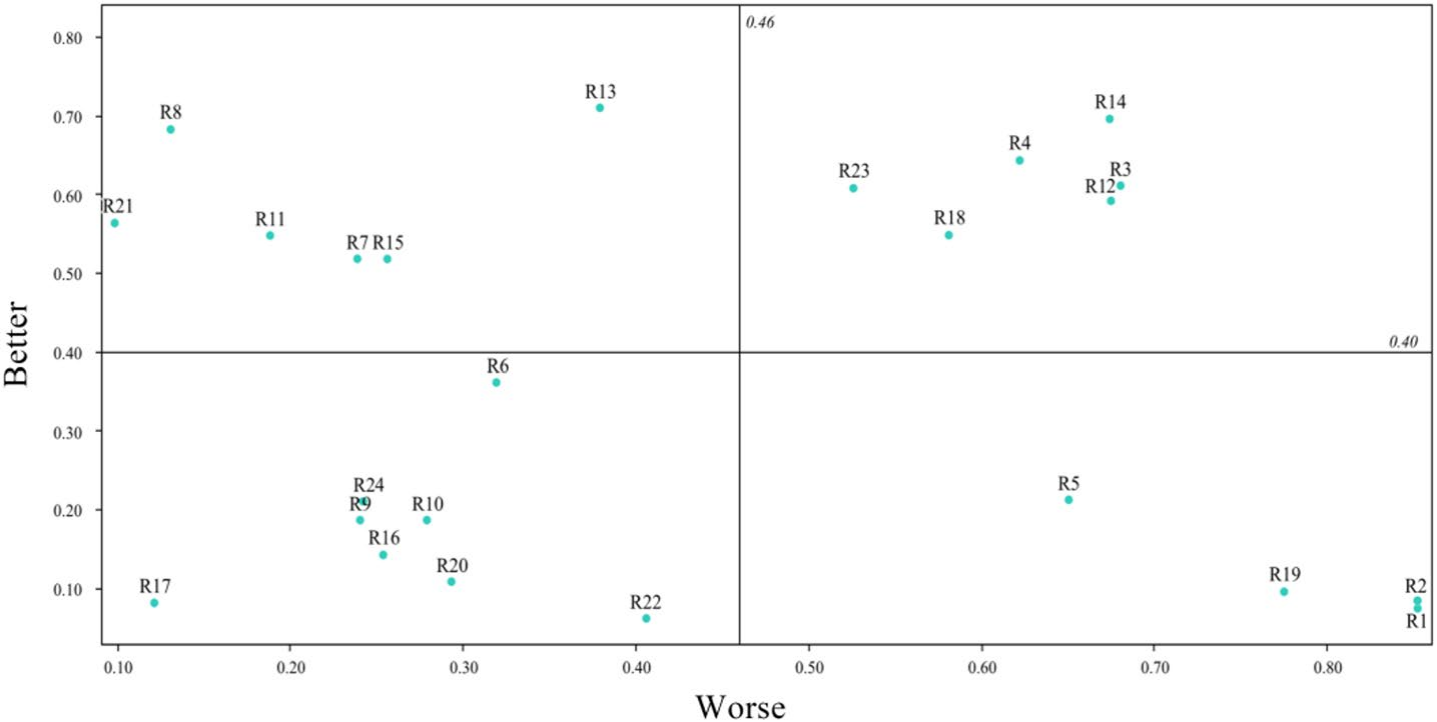
Better-Worse coefficient graph.

In order to have a more intuitive understanding of users’ selection tendencies for the design requirements of one-piece ski suits, a scatter plot of the Better-Worse coefficient was drawn, as shown in Fig. 3. Through the statistics and processing of the table data, a matrix diagram of 24 demand indicators was drawn. Among them, the Better coefficient was taken as the X-axis, the Worse coefficient was taken as the Y-axis with the absolute value of the data, the average value of the Better coefficient was taken as the center line of the X-axis, and the average value of the Worse coefficient was taken as the center line of the Y-axis. The quartile plot of the Better-Worse coefficient is shown in Fig. 3.

As can be analyzed from Fig. 3, in terms of user requirements, the absolute values of the Better and Worse values of the four requirement items R3, R4, R12, and R14 in the one-dimensional quality requirement (the first quadrant) are relatively high. Prioritizing their satisfaction in subsequent design and development will help significantly improve user satisfaction. In the attractive quality requirement (the second quadrant), the Better values of the R8, R11, and R21 requirements are high, while the absolute values of the Worse values are low. If these requirements are not met, user satisfaction will not decrease, but if they are met, user satisfaction will increase significantly. In the design, they should be met as much as possible. Indifferent quality requirement (the third quadrant), with a low Better value and a low absolute value of the Worse value, such requirements are usually not provided. In the must-be quality requirement (the fourth quadrant), the Better values of the three demand items R1, R2, and R19 are low, while the absolute values of the Worse values are high. These are the basic functions that consumers consider essential and must be met in the design. When formulating the design strategy for one-piece ski suits, in terms of user demands, on the basis of meeting the must-be quality requirement, priority should be given to meeting the one-dimensional quality requirement and the attractive quality requirement.

## Construction of design strategy based on AHP

### Establish a hierarchical structure model

**Fig. 4:**
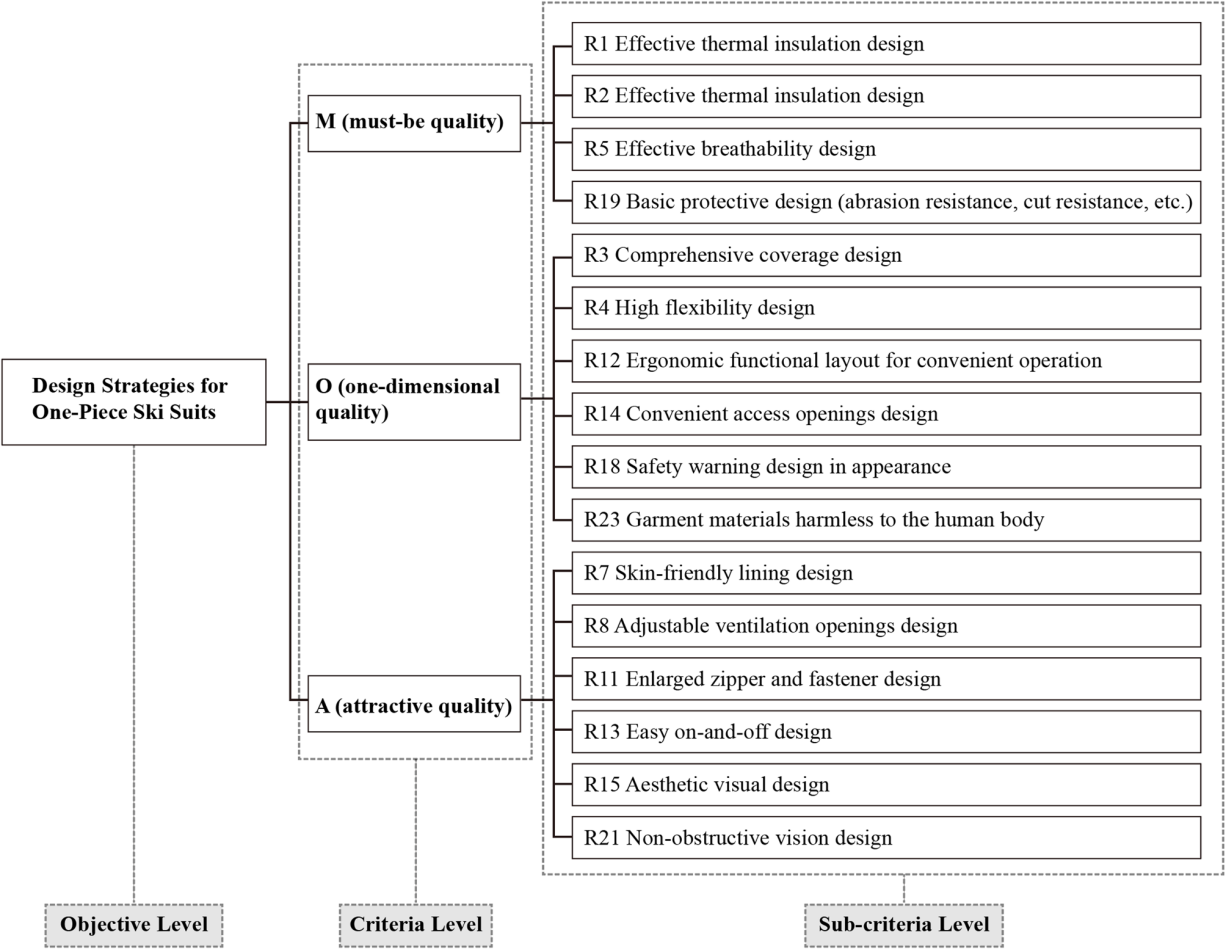
Hierarchical structure model of demand elements for one-piece ski suits.

Based on the user demand attributes and user demand quadrant diagrams of one-piece ski suits summarized by the Kano model, combined with expert interviews, a hierarchical model of design demands for one-piece ski suits is constructed. Determine the objective level according to the category as the problem to be solved, that is the design strategies of one-piece ski suits, and the criterion level as the conditions required to achieve the objective level, that is the design requirements of the three attributes M, O and A. The sub-criterion level is an extension of the criterion level, namely the specific design elements of one-piece ski suits. The construction of the demand element hierarchical structure model for one-piece ski suits is shown in Fig. 4.

### Calculation of demand element weights based on AHP

In order to establish the exact weights of each design element and ensure its rigor and rationality, the AHP questionnaire with a 9-level scale was used to conduct the research on expert evaluation. The expert team consists of 15 professionals involved in or designing ski clothing, including 1 professional skier, 3 professional ski clothing designers, 6 teachers specializing in fashion design, and 5 master’s and doctoral students majoring in fashion design. The athlete expert has rich wearing experience of one-piece ski suits, and the other experts have all participated in multiple ski suit design and relevant product development projects, with rich design experience. First, the expert team is invited to compare and evaluate the importance of each requirement in the criterion level and sub-criterion level of the hierarchical structure model in a pairwise manner. Then, normalization processing is carried out and the weights of each design requirement are obtained. Finally, the results are subjected to consistency verification. The consistency verification formula is shown in Eq. (3). $\:RI$ is the average random consistency index, $\:{\lambda\:}_{\mathrm{m}\mathrm{a}\mathrm{x}}$ is the maximum eigenvalue of the judgment matrix, $\:n$ is the order of the matrix, and $\:CR$ < 0.1 indicates that the analysis process is reasonable.

3 $$\:CR=\frac{{\lambda\:}_{\mathrm{m}\mathrm{a}\mathrm{x}}-n}{\left(n-1\right)\times\:RI}$$

The importance of the classification of design requirements at the criterion level was compared and scored, and a weight judgment matrix for the design requirement types of one-piece ski suits was constructed, as shown in Table 6. According to the calculation of Eq. (3), it can be known that $\:CR=0.00876$. Therefore, this weight judgment matrix passes the consistency test.

**Table 6:** Weight judgment matrix for the types of design requirements of one-piece ski suits.

	M	O	A	Weighting
M	1	2	3	53.8961%
O	1/2	1	2	29.72583%
A	1/3	1/2	1	16.37807%

Note: M: Must-Be Quality; O: One-Dimensional Quality; A: Attractive Quality.

The importance of the 16 specific design requirement elements of one-piece ski suits in the sub-criterion level was compared and scored, and the secondary requirement weights under the three requirement types of M, O, and A were calculated respectively. According to the calculation of Eq. (3), it can be known that $\:CR$ in the M attribute index matrix is 0.01171, $\:CR$ in the O attribute index matrix is 0.05713, and $\:CR$ in the A attribute index matrix is 0.0324, all of which pass the consistency test. Based on the judgment matrix constructed in Table 6, all secondary requirement weights were normalized. Finally, the comprehensive weights and rankings of each design requirement element were obtained, as shown in Table 7.

**Table 7:** Comprehensive weights and rankings of design requirement elements for one-piece ski suits.

Requirement Type	Requirement type weights	Sub-criteria level	Sub-criteria level requirement weights	Combined sub-criteria level requirement weights	Combined weights
M	53.8961%	R1 Effective thermal insulation design	46.58194%	25.10585%	1
		R2 Effective wind resistance design	27.71405%	14.93679%	2
		R5 Effective breathability design	16.10702%	8.68106%	4
		R19 Basic protective design (abrasion resistance, cut resistance, etc.)	9.59699%	5.1724%	7
O	29.72583%	R3 Comprehensive coverage design	26.89134%	7.99367%	5
		R4 High flexibility design	15.82669%	4.70461%	9
		R12 Ergonomic functional layout for convenient operation	11.98123%	3.56152%	10
		R14 Convenient access openings design	35.21187%	10.46702%	3
		R18 Safety warning design in appearance	3.61297%	1.07398%	14
		R23 Garment materials harmless to the human body	6.4759%	1.92502%	12
A	16.37807%	R7 Skin-friendly lining design	5.90886%	0.96776%	15
		R8 Adjustable ventilation openings design	36.74345%	6.01787%	6
		R11 Enlarged zipper and fastener design	4.31857%	0.7073%	16
		R13 Easy on-and-off design	28.96395%	4.74373%	8
		R15 Aesthetic visual design	14.58455%	2.38867%	11
		R21 Non-obstructive vision design	9.48063%	1.55274%	13

Note: M: Must-Be Quality; O: One-Dimensional Quality; A: Attractive Quality.

When designing one-piece ski suits, quantitatively analyzing the demand elements of the wearer is an important means to assist in design decisions. By comparing the weights of the importance of each demand indicator and giving priority to meeting the demands with higher weights within the limited design resources, the success rate of the design of this ski suit can be effectively improved.

### Analysis of design element weights and design strategies for one-piece ski suits

According to the research results, it can be known that the users’ demands for one-piece ski suits are mostly essential attributes and demand items with high weights in terms of basic performance, and there are also certain demand item distributions with high weights in terms of structural design and safety performance design. The demand items in terms of appearance design can help enhance the user experience, but they are not the main concern of users. The demand items related to users’ health in sustainable design are particularly favored by users, while other factors receive less attention, and the application of recycled materials may reduce users’ favorability. Specific analysis of design elements is as follows:

Among the Must-be Quality (M), R1 (Effective thermal insulation design), R2 (Effective wind resistance design) and R5 (Effective breathability design), which are core requirements, are not only the core focus factors for users to choose ski suits, but also the key directions for enterprises in ski suit product design. As the basic performance of ski suits, thermal insulation, wind resistance and breathability are mainly related to material selection and style & structure design. The selected material performance should meet the needs of thermal insulation and wind resistance from multiple aspects including outer fabric, filling material and inner fabric, while ensuring breathability; in terms of style and structure, the characteristics of the one-piece style should be utilized, and loop structures should be designed at parts such as cuffs, trouser leg openings and necklines to adjust the balance between wind resistance, thermal insulation and breathability, and active ventilation openings should be set at areas prone to heat accumulation such as armpits and chest. R19 (Basic protective design (abrasion resistance, cut resistance, etc.)) is also a requirement item with high comprehensive weight, which can be mainly improved through the selection of high-strength materials for garments and the design of thickened wear-resistant structures.

High completion degree of One-dimensional Quality (O) can quickly improve user satisfaction, while the opposite will lead to a decrease in the satisfaction of the target product. R14 (Convenient access openings design) is the core requirement among expected requirements and a key design object; it is a user pain point caused by the style of one-piece ski suits and should be prioritized to meet through methods such as structural design. Meanwhile, R3 (Comprehensive coverage design) is also a key design element. The one-piece ski suit style itself has relatively good torso coverage; on this basis, focusing on the structural design of cuffs and trouser openings to avoid unnecessary displacement of the garment can further improve this aspect. The two requirements of R4 (High flexibility design) and R12 (Ergonomic functional layout for convenient operation) are mainly related to style & structure design, and can be improved through the design of garment styles, quality control, and considerations of garment cutting and functional layout methods. R18 (Safety warning design in appearance) and R23 (Garment materials harmless to the human body) have low user demand degree, but they are related to users’ personal safety, so their importance cannot be ignored.

The fulfillment of Attractive Quality (A) can bring surprises to users. R8 (Adjustable ventilation openings design) and R13 (Easy on-and-off design) have relatively high comprehensive demand weight, and are mainly realized through the structural design of the garment. Meanwhile, R15 (Aesthetic visual design) is an important factor affecting users’ selection of ski suit products and also an important means for brand differentiated competition, which should be emphasized while meeting garment performance. Failing to meet Attractive Quality requirements will not reduce user satisfaction; therefore, R7 (Skin-friendly lining design), R11 (Enlarged zipper and fastener design) and R21 (Non-obstructive vision design) are respectively related to detailed designs that improve garment texture, garment convenience and safety. As elements with relatively low comprehensive demand weight, they are not regarded as necessary development content under the premise of limited design resources. However, in the development of high-end products, these details are often used to highlight brand characteristics and enhance user stickiness, and are one of the key design contents. The design of high-end products should not only focus on garment performance but also consider the detailed designs that can bring surprises to users.

Notably, although Indifferent Quality (I) and Reverse Quality (R) receive low attention from users and may cause disgust, R22 (High durability of garment materials) and R24 (Use of recycled sustainable materials) among them are attributes that indirectly or directly improve garment sustainability. Experts point out that these attributes have positive significance for the development of the heavily polluting textile and garment industry. Therefore, they cannot be ignored in development, and can be promoted for users to gradually accept through methods such as design empowerment and brand concept guidance.

According to the above analysis, the design of one-piece ski suits should start from multiple dimensions, fully consider their actual needs in the usage environment, and meet users’ design expectations to optimize the wearing experience. Therefore, the design strategy of one-piece ski suits driven by demand is as follows:

1. Possess the basic performance of effective ski suits. This garment should have excellent warmth retention, wind resistance, breathability, coverage and strong flexibility design. By choosing advanced insulation, waterproof, windproof materials and breathable layers, as well as improving the sealing design of the clothing, it is ensured that users remain warm, dry and comfortable in bad weather conditions. By taking advantage of the one-piece style, the style design is combined with the application of elastic materials to ensure that the clothing covers a large area of the user’s body and provides freedom during movement.
2. Possess a reasonable and convenient style and structure design. The functional design and layout of this garment should conform to ergonomics. The application of structural design makes it convenient, fast and comfortable for users to interact with the garment. By means of three-dimensional cutting and flexible application of clothing structure and accessories, the pain point of inconvenience in putting on and taking off one-piece styles is solved. Adjustable breathable openings are designed to cope with dynamic wearing environments. According to the actual application situation, use lining with good skin affinity as appropriate to enhance the comfort of the contact area between the skin and the clothing. The enlarged zipper and loop design facilitate users to wear gloves for operation.
3. Possess a comprehensive security design. This garment should have protective properties such as anti-scratch and anti-cut to provide basic protection for users. Warning colors or reflective designs should be set in obvious places to be recognizable by viewers under limited visibility conditions. Structures such as drawstrings and adjustment loops should be used to ensure that the collar and cap of the garment do not block the wearer’s view.
4. Possess a certain degree of aesthetic visual design. This clothing should have a relatively good visual effect, enhancing the wearer’s image while adding certain social attributes to them, thereby improving the market competitiveness of the product.
5. Have sustainable design and guidance. The material selection of this clothing should be non-toxic and harmless to the wearer to ensure their health. Under this premise, it is called on the wearer to use materials with sustainable properties within the range they can accept.

## Design and evaluation of one-piece ski suits

### One-piece ski suit design scheme

**Fig. 5:**
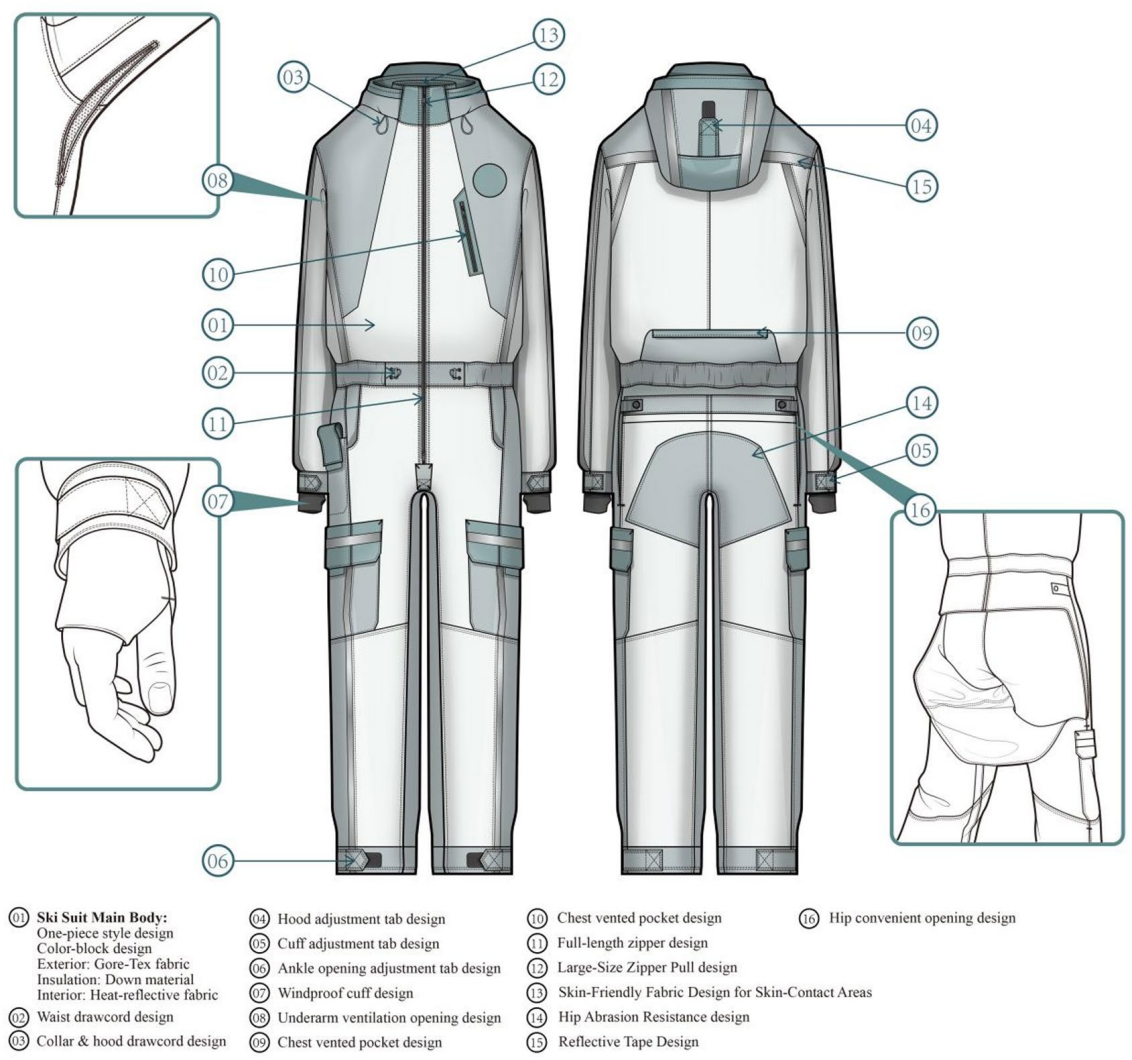
Design effect drawing of the one-piece ski suit.

Based on the design strategy of one-piece ski suits proposed in the previous research, the design and development plan of one-piece ski suits is carried out, as shown in Fig. 5. The specific design plan is: (1) In terms of style and structural design, the one-piece ski suit style is adopted. The front of the door is designed with a double-zipper running through to ensure its convenience in putting on and taking off. The hood is designed with drawstrings and loop structures to adjust the wind resistance and warmth retention of the cap and its obstruction of the lateral view. The collar and trouser cuffs are designed with loop structures to balance the breathability and wind resistance of the clothing. The cuffs are designed with windproof sleeves to prevent unnecessary displacement of the sleeves due to large movements. The waist is designed with a drawstring structure to adjust the fit of the garment. (2) In terms of appearance and color, the overall appearance design combines and echoes the function of the clothing with its structural appearance. It uses a color-blocking design and places reflective strips in multiple locations, enhancing the visibility of the clothing while maintaining its aesthetic appeal. (3) In terms of material selection, Gore-Tex fabric is planned to be selected for outer layer material to ensure wind resistance and certain breathability and wear resistance. Down filling material is planned to be used to ensure the warmth and flexibility of the clothing. In the main areas, heat-reflective fabric is planned to be used to improve the warmth of the clothing. Fleece fabric is planned to be used on the inner side of the collar and the inner side of the cuff to ensure the personal wearability of the clothing and the skin contact area. (4) In terms of functional design, the buttocks of the clothing are designed with convenient openings for hand release and use a loop structure for privacy design. The armpits and lower back are designed with breathable openings, and the chest pockets are designed with mesh bag fabric to adjust the breathability of the clothing. Large-sized pullers, fasteners, and loop designs are used to facilitate users’ operation while wearing gloves.

The design scheme is carried out from 4 aspects including the three garment elements style, color, material and functional design, based on the one-piece ski suit design strategies proposed in the above research. The corresponding relationships between specific elements in the design scheme and design strategies are shown in Table 8.

**Table 8:** Correspondence between schemes and design Strategies.

Corresponding Strategy	Specific Strategy	Corresponding Design Elements	Design Objective
Basic Performance	Windproof and Warmth Design	Select one-piece style (Fig. 5.01)	The waist of the one-piece structure has good warmth retention
		The outer layer uses Gore-Tex material, selection of down filling material, selection of internal thermal reflective fabric (Fig. 5.01)	Improve windproof and thermal insulation efficiency in multiple dimensions
		Drawstring and buckle structure (Fig. 5. 03、05、06)	Adjust windproof and warmth performance
		Windproof cuff design (Fig. 5.07)	Prevent cold air from entering the cuffs
	Breathability Design	The outer layer uses Gore-Tex material (Fig. 5.01)	Ensure certain breathability while guaranteeing windproof performance
		Breathable opening design at armpits and lower back, breathable pocket design at chest (Fig. 5. 08、09、10)	Breathability design in heat-concentrated areas
		Drawstring and buckle structure (Fig. 5. 03、05、06)	Adjust breathability
	Coverage Design	Select one-piece style (Fig. 5.01)	The waist has good coverage
		Windproof cuff design (Fig. 5.07)	Avoid cuff displacement during movement
	Flexibility Design	Select one-piece style (Fig. 5.01)	The ski suit structure is simple and flexible
		Use down filling material(Fig. 5.01)	The ski suit is light in weight
		Drawstring structure(Fig. 5.02)	Ensure the fit degree of the ski suit
Structural Design	Convenience Design	Hip Convenient Opening Design (Fig. 5.16)	Facilitate wearers to relieve themselves and solve the pain point of one-piece style
		Double-headed zipper through design (Fig. 5.11)	Increase the convenience of putting on and taking off
		Design of large-size zipper sliders, fasteners and buckles (Fig. 5. 02、03、04、05、06、12)	Facilitate users to operate with gloves
	Adjustable Design	Each breathable opening uses zipper opening and closing (Fig. 5. 03、05、06、08、09、10)	Adjust the ski suit’s breathability according to user needs
	Skin-friendly Design	Use fleece fabric at parts in contact with skin (Fig. 5.13)	Materials with low thermal conductivity reduce users’ cold touch sensation
Aesthetic Design	Visual Effect Design	Color-blocking design (Fig. 5.01)	Enhance the layering of appearance design
		Combination of function and segmented structure (Fig. 5.01)	Enhance the aesthetic degree of the ski suit
Safety Performance	Basic Protective Design	The outer layer uses Gore-Tex material, and abrasion-resistant design is adopted for the buttocks (Fig. 5. 01、10)	The outer fabric has relatively high strength, and abrasion-resistant design is adopted for easily worn parts
	Recognizability Design	Color selection and reflective strip design (Fig. 5.01 、15)	Increase visibility in skiing environments
	Non-obstructive Vision Design	Hood, neck drawstring and rear adjustment loop (Fig. 5. 03、04)	Adjust the ski suit shape to avoid blocking the view
Sustainability	Material Selection and Sustainable Concept Guidance	Application of Gore-Tex material and technical fabric (Fig. 5.01)	Improve the ski suit’s thermal insulation efficiency and indirectly reduce unnecessary material waste

### Design evaluation

Users’ evaluations of the design of one-piece ski suits vary from person to person. To ensure the objectivity of the evaluation results, combined with the weights of the design requirement indicators obtained from the research results of Kano-AHP, the Fuzzy Comprehensive Evaluation Method is used to evaluate the design scheme, and 15 experts who participated in the AHP expert survey are invited to score and evaluate the above design scheme in an online form. The specific steps are as follows:

1. Set the evaluation grade of the one-piece ski suit and assign the corresponding scores in sequence: excellent (90 points), good (80 points), qualified (60 points), poor (40 points), very poor (20 points). Therefore, the evaluation grade vector is $\:V=\left[\mathrm{90,80,60,40,20}\right]\:$.

2) Organize an expert team to grade and score the design requirement indicators retained at the sub-criterion level, and establish a fuzzy comprehensive evaluation matrix. The results are as follows:

$$\:{R}_{1}=\left[\begin{array}{ccccc}0.533&\:0.333&\:0.133&\:0.000&\:0.000\\\:0.600&\:0.333&\:0.067&\:0.000&\:0.000\\\:0.533&\:0.267&\:0.200&\:0.000&\:0.000\\\:0.667&\:0.200&\:0.067&\:0.067&\:0.000\\\:0.800&\:0.133&\:0.067&\:0.000&\:0.000\\\:0.533&\:0.467&\:0.000&\:0.000&\:0.000\\\:0.267&\:0.200&\:0.400&\:0.133&\:0.000\\\:0.267&\:0.200&\:0.400&\:0.067&\:0.067\\\:0.400&\:0.400&\:0.200&\:0.000&\:0.000\\\:0.333&\:0.200&\:0.400&\:0.067&\:0.000\\\:0.467&\:0.400&\:0.067&\:0.067&\:0.000\\\:0.200&\:0.533&\:0.267&\:0.000&\:0.000\\\:0.467&\:0.267&\:0.133&\:0.000&\:0.000\\\:0.667&\:0.200&\:0.133&\:0.000&\:0.000\\\:0.400&\:0.333&\:0.200&\:0.067&\:0.000\\\:0.733&\:0.200&\:0.067&\:0.000&\:0.000\end{array}\right]$$

3) Calculate the comprehensive evaluation vector of the design requirements for one-piece ski suits. The calculation formula is shown in Eq. 4:

4 $$\:{B}_{R}=A{\times}R$$

Type, $\:A$ as evaluation index weight, namely Table 6 by AHP method have been the son needs comprehensive weighting criterion level, thus $\:{B}_{R}=\left[\mathrm{0.531,0.293,0.150,0.022,0.004}\right]$.

4) The final evaluation score is calculated based on the scoring grade. The calculation formula is shown in Eq. 5:

5 $$\:P={B}_{R}{\times}V$$

Thus, the final evaluation score of $\:P=\left[\mathrm{0.531,0.293,0.150,0.022,0.004}\right]\times\:\left[\mathrm{90,80,60,40,20}\right]=81.193$.

According to the Fuzzy Comprehensive Evaluation results, the one-piece ski suit scheme designed based on the one-piece ski suit design strategy proposed by analyzing and researching with the Kano-AHP method finally has a fuzzy evaluation score in the range of 80–90 points, with a good comprehensive rating. This design scheme can meet users’ design needs to a certain extent and improve users’ wearing experience.

## Conclusion

This study aims to explore the design methods of one-piece ski suits based on user needs. First of all, collect the design requirements of one-piece ski suits through user interviews and user journey map; Secondly, the design requirements are qualitatively analyzed through the Kano model. The attribute indicators of M, O and A are retained, while the attribute indicators of I and R are excluded. Again, the weight values of the M, O and A attribute indicators are quantitatively calculated through the AHP method; Finally, it is transformed into a design strategy through the hierarchical sorting of design requirements and verified by examples. The results indicate that through this method, a clothing functional design model centered on user needs is constructed, transforming the subjective judgment in clothing product design into a more scientific and objective decision-making process, and providing certain theoretical and practical support for design decisions. However, this study still has certain limitations. The current research mainly focuses on the conceptual level of the design strategy for one-piece ski suits. In the actual design and production, multiple factors such as production costs and material properties still need to be considered to ensure the reliability and practical feasibility of the design results. Future research should integrate more factors and be committed to constructing a more complete design strategy system, so that the design results are more closely combined with the actual industrial needs.

## Data Availability

Data are contained within the article.
